# Can Machine Learning Be Better than Biased Readers?

**DOI:** 10.3390/tomography9030074

**Published:** 2023-04-28

**Authors:** Atsuhiro Hibi, Rui Zhu, Pascal N. Tyrrell

**Affiliations:** 1 Institute of Medical Science, University of Toronto, Toronto, ON M5S 1A8, Canada; 2 Department of Medical Imaging, University of Toronto, Toronto, ON M5T 1W7, Canada; 3 Department of Computer Science, University of Toronto, Toronto, ON M5S 2E4, Canada; 4 Department of Statistical Sciences, University of Toronto, Toronto, ON M5G 1Z5, Canada

**Keywords:** machine learning, annotation bias, labeling consensus, chest X-ray, convolutional neural network

## Abstract

**Background:** Training machine learning (ML) models in medical imaging requires large amounts of labeled data. To minimize labeling workload, it is common to divide training data among multiple readers for separate annotation without consensus and then combine the labeled data for training a ML model. This can lead to a biased training dataset and poor ML algorithm prediction performance. The purpose of this study is to determine if ML algorithms can overcome biases caused by multiple readers’ labeling without consensus. **Methods:** This study used a publicly available chest X-ray dataset of pediatric pneumonia. As an analogy to a practical dataset without labeling consensus among multiple readers, random and systematic errors were artificially added to the dataset to generate biased data for a binary-class classification task. The Resnet18-based convolutional neural network (CNN) was used as a baseline model. A Resnet18 model with a regularization term added as a loss function was utilized to examine for improvement in the baseline model. **Results:** The effects of false positive labels, false negative labels, and random errors (5–25%) resulted in a loss of AUC (0–14%) when training a binary CNN classifier. The model with a regularized loss function improved the AUC (75–84%) over that of the baseline model (65–79%). **Conclusion:** This study indicated that it is possible for ML algorithms to overcome individual readers’ biases when consensus is not available. It is recommended to use regularized loss functions when allocating annotation tasks to multiple readers as they are easy to implement and effective in mitigating biased labels.

## 1. Introduction

The availability of great computing power and large amounts of medical imaging data have allowed machine learning (ML) to assist radiologists when making a diagnosis. One of the most recent applications is image classification using supervised learning algorithms, which require a large amount of expert-labeled medical image data. However, the labeling process can be a time-consuming and laborious work for many experts [1]. 

In many practical settings, as a single expert cannot deal with enormous amounts of data, it is common to divide training data among multiple readers to alleviate the labeling workload. Another common situation is when combining labeled datasets from different sources/institutions. The multiple datasets labeled by different readers are combined for training. However, labeled data from multiple readers can generate biased labels for various reasons, such as variance in expertise, prior information provided by other readers/clinicians, and ambiguous pixels [2,3]. In addition to these systematic errors, random errors can also occur among readers due to increased fatigue, for example [3,4]

Although achieving a perfect gold standard in medical imaging is often challenging [5], labels that can reach consensus among readers can be seen as a better imperfect gold standard than individual readers’ annotations because biases caused by readers’ background and random errors could be reduced in labels agreed among multiple readers. However, reviewing other readers’ labels and coming to an agreement through discussions can be costly and time-consuming for readers. Recently, some studies [6,7] proposed ways to construct machine learning models for noisy datasets, but it is still unknown if ML models can amend for the biases caused by multiple readers.

The purpose of this study is to determine if ML algorithms can overcome the biases generated by multiple readers and more closely match a consensus than individual readers. The objectives of this study are as follows: (1) demonstrate that a ML model trained on datasets labeled by multiple readers with biases can closely match the consensus of the readers than individual readers who have biases; (2) compare the model performances by introducing some artificial biases while varying their strength; and (3) introduce a robust loss function to reduce the effect of biases.

## 2. Materials and Methods

### 2.1. Datasets

The experiments of this study were conducted using a binary-class dataset of chest X-ray images of pediatric pneumonia [8]. We chose the chest X-ray dataset since it is one of the medical imaging modalities in which reading errors have been reported in a previous study [3]. The labels which come with the dataset were assumed to be the consensus of the simulated readers, as they are the closest labels that we could obtain to the ground-truth labels. The chest X-ray dataset of pediatric pneumonia was collected from the Guangzhou Women and Children’s Medical Center, consisting of a total of 5863 images. The original training set included 3883 pneumonia images (2538 bacterial and 1345 viral) and 1349 normal images. The original test set included 234 normal images and 390 pneumonia images. To resolve the class imbalance issue in the training set due to the lack of normal images and reduce the time needed for training, 500 normal images and 500 pneumonia images were randomly sampled from the original training set as a new training set: 109 normal images and 109 pneumonia images were used for validation, and 234 normal images and 390 pneumonia images were used for testing the model.

### 2.2. Problem Setting

In this study, we focused on the following two types of errors that could be made by multiple readers during their labeling process:

1. Random error: readers accidentally introduce wrong labels.
2. Systematic error: readers make mistakes when interpreting an image due to a bias.

Random errors could happen when readers experience increased symptoms of fatigue and oculomotor strain, resulting in reduced ability to detect abnormalities [3,4]. Systematic errors could occur due to variance in radiological experience, prior information provided by another clinician/reader, or even a difference in viewing environment (e.g., poor lighting conditions) [3]. We called readers who made systematic errors *biased readers*. In this study, two kinds of labeling generated based on systematic errors were introduced: false positive labels (original negative labels were treated as positive labels) and false negative labels (original positive were treated as negative labels). Bias was introduced to a percentage (5–25%) of randomly selected (without replacement) labels. The strength of the random and systematic errors applied to the training dataset is shown in Table 1. For a set of input images, we assumed they were assigned with biased labels by multiple readers and the ground-truth labels were not available during the training phase. It was also assumed that each reader’s labeling work was equally allocated.

### 2.3. Model and Loss Functions

In this study, we employed the convolutional neural network (CNN) architecture of Resnet18 [9] with a two-class output in the last layer. The Resnet18 has been used in a previous study [10] and can save training time as it has a relatively small architecture. In our experiment, a baseline model and an improved model were trained on the datasets with different kinds of reader biases introduced. The former has the architecture of Resnet18 with a cross-entropy loss $L_{cross\_entropy}$. The latter has the Resnet18 architecture with $L_{cross\_entropy}$ and a paired softmax divergence regularization (PSDR) loss $L_{PSDR}$ [7], that is, the total loss function is a sum of these two functions,

$$L_{cross\_entropy}+\alpha L_{PSDR}$$

with a hyperparameter α. The PSDR loss, originally proposed by Chen et al. [7], is defined as follows:

$$L_{PSDR}(x;\theta )=\sum_{t}KL(f(x_{t}^{\prime };\theta )||f\left(x_{t}^{\prime \prime };\theta \right),$$

where $f\left(x;\theta \right)$ denotes a neural network parameterized by θ, which predicts a probability distribution over all classes for any input image x, and $x_{t}^{\prime }$ and $x_{t}^{\prime \prime }$ are generated from random data augmentation of the original image $x_{t}$. Kullback–Leibler (KL) divergence is denoted by KL(⋅), which measures the similarity between the two outputs. The PSDR loss that serves as a regularization term consists of the sum of KL divergence between the output of two images randomly generated from the original image using data augmentation. The PSDR loss is known to provide a powerful and simple way for improving robustness to various kinds of noise in a training dataset, and it can be used as a supplementary to the cross-entropy loss [7].

In the context of PSRD loss, applying a wide variety of data augmentation techniques is recommended so that the regularization term of the PSDR loss is able to explicitly penalize the difference between the predictions from paired samples generated by data augmentation. Therefore, in our study, horizontal random flipping and vertical random flipping were both applied to the training data as data augmentation. However, in an applied study, it is often advisable to maintain anatomical symmetry when applying augmentation techniques, and this should be verified. It should be noted that data augmentation was only applied to the training dataset; the original test set was left unchanged.

### 2.4. Environment and Implementation Details

The experiments were run using a Python 3.7 interpreter on a machine with one RTX2080 GPU and an AMD Ryzen 5 3600X 6-Core Processor as the CPU. The codes were implemented using Pytorch.

The optimizer used for training was Adam with a learning rate of 1 × 10^−3^. The training went for 50 epochs, and the training was stopped at the point when no improvement in training accuracy and training loss was observed. The hyperparameter α in the PSDR loss function was tuned using a clean validation set every time the model was trained. During the preprocessing phase, we applied resizing and normalization to each input image. Resizing of chest X-ray images to (224, 224, 224) pixels was performed to fit the input shape of Resnet18. After that, we normalized the input images with mean of 0.5 and a standard deviation of 0.2365, as recommended in a previous study [9].

### 2.5. Experimental Methodology

The metric used for evaluating reader performance and model performance was the area under ROC curve score (AUC). Different biases were introduced to the dataset to investigate how they affected the models’ test AUC. For each bias and each model, the following steps were performed: (1) introduce readers’ biases to the training labels and validation labels to generate a new training set and a validation set with biased labels; (2) train the model on the biased training set. The hyperparameter was tuned using the validation dataset; (3) evaluate the models on the test set; and (4) repeat steps 2 and 3 for 5 repetitions (seed = 42).

To investigate the differences in the models’ AUC slopes depending on the type and strength of biases, descriptive statistics were performed. The means with standard deviations were provided. A comparison of the means was tested with ANCOVA using R (version 4.2.2).

## 3. Results

**Figure 1:**
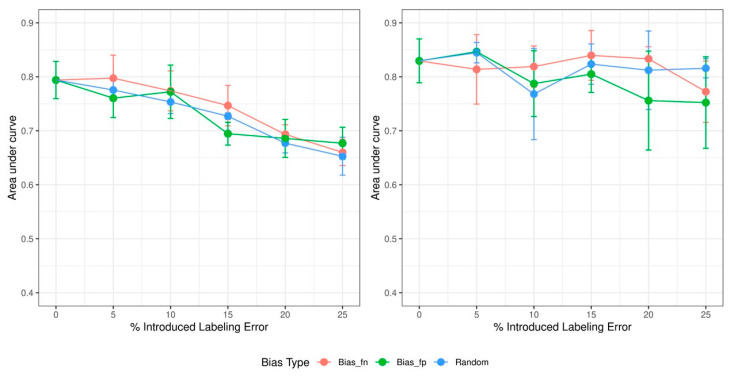
The trend of the AUC in the baseline model (**left**) and the improved model (**right**). *Bias_fn* is systematic error with false negative labeling. *Bias_fp* indicates systematic error with false positive labeling. *Random* is random error.

We conducted experiments on each combination of bias strength and type for five repeats. The hyperparameter was tuned on a clean validation set every time the model was trained. The AUC observed in the baseline model and the improved model is depicted in Figure 1. The vertical axis indicates the AUC, and the horizontal axis shows the percentage of the introduced labeling error. The error bars show the standard deviations of the AUC with respect to five repeats. It is observed that, as the percentage of overall error increases, the baseline model’s AUC decreases, whereas the improved model with the PSDR loss is less affected.

The statistical analysis indicated that the differences in the models’ AUC slopes between false negative and false positive biases for both models are not statistically significant (false positive: β = −0.005 with *p* = 0.005; false negative: β = −0.0045 with *p* = 0.002; and random error: β = −0.0043 with *p* = 0.0002. The difference between the slopes: *p* = 0.74 for the baseline model; false positive: β = −0.0037 with *p* = 0.02; false negative: β = −0.0012 with *p* = 0.36; and random error: β = −0.0006 with *p* = 0.66. The difference between the slopes: *p* = 0.18 for the improved model). All descriptive statistics of the models trained using different strengths and types of biases are shown in Supplementary Table S1.

**Figure 2:**
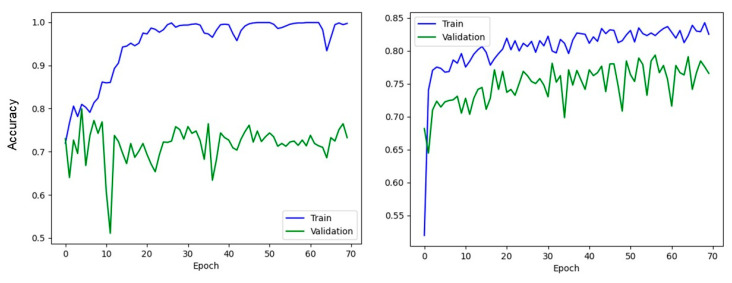
Training and validation accuracy throughout the training process for both models when the training set contains 15% random error: (**left**) the baseline model and (**right**) the improved model.

It is also observed from Figure 1 that as the AUC decreases due to bias, the baseline model’s accuracy decreases. This is mainly due to overfitting, when the model learns the training data so well that it fails to generalize to unseen data. The training and validation accuracy throughout the training process is illustrated in Figure 2. The figure indicates that the gap between training and validation accuracy is larger in the baseline model than that in the improved model.

## 4. Discussion

Our study demonstrated that a regularized model achieved a higher AUC than individual readers with biases and closely matched the consensus with a higher AUC in most cases. This implied that the effect of biased labels that could inevitably happen among multiple readers could be reduced solely by adding regularizing terms when constructing ML-based classification models. This simple but effective approach could significantly help ML-driven medical imaging studies that require large datasets without labeling consensus. 

### 4.1. Dose Effect of Increasing Errors

In this study, we focused on two types of error: random error and systematic error. A random error occurs when readers accidentally assign wrong labels, whereas a systematic error can be introduced when readers interpret an image in a different, planned way. As seen in the results provided through our analysis, systematic errors lead to models with lower predictability. This is a limitation when developing ML models in medical imaging, especially for improving diagnostic accuracy to support healthcare professionals [11]. A previous study led by Tanno et al. [6] proposed a regularized loss function applicable to biased labels, but it did not investigate the effects of type and strength of biases. Our study systematically analyzed various types and strengths of errors and evaluated their effects on classification performance in a widely used medical imaging dataset. 

It can be observed in Figure 1 that increasing bias results in decreased AUC of the ML binary classifier models and that a model using regularized loss function is less influenced by labeling bias. This is supported by the trend shown in the training and validation accuracy. As shown in Figure 2, training in the baseline model does not enhance validation accuracy and results in a larger gap between training and validation accuracy compared to the model with the PSDR regularization loss. This is a typical phenomenon observed in overfitting cases. Since the regularization loss encourages the model to learn a smoother decision boundary and is less likely to be influenced by outliers, the model with the PSDR loss could achieve more robust predictions than the baseline model.

### 4.2. Effects of Different Types of Biases

In this study, in addition to random errors, two kinds of labeling generated based on systematic errors were introduced: false positive labels and false negative labels. The differences in the fitted slopes of both models’ AUC for different bias types are not statistically significant. When researchers request multiple readers to label datasets for developing ML models, the types and strengths of biases are not known in advance. This reinforces the usefulness of the regularized loss function in these circumstances as it is shown to be useful in alleviating a wide range of types and strengths of labeling errors. In the situation where a training dataset is labeled by multiple readers with different medical expertise and the training dataset contains a single type of bias, either false negative or false positive labels, as described in Figure 1, labels with a small number of systematic errors (not shared by all readers) could be corrected by adopting the regularized loss function in many cases. However, in order to make sure that these small amounts of single-sided biases are successfully corrected, an additional evaluation is needed by using an external test dataset when deploying ML models in medical settings. Another possible situation is where a similar bias is equally shared by all readers caused by, for example, ambiguous pixels around lesions [2]. In this case, training a model will be successful, but it will be challenging (near impossible) to correct for the biased labels as the resulting model will be accurate but biased. An evaluation on an external test dataset is again effective to assess generalizability and identify the bias.

### 4.3. Limitations

Although this study managed to show how ML algorithms could reduce the effect of biases and errors made by human readers, there were some limitations. First, the bias introduced in this study was artificial and might not fully represent a real-life observer error. Further study would be needed using a dataset in which the findings could be related to specific artifact or improper exposure, or other technical features. The second limitation was the number of datasets used in the analysis. Since we only used a chest X-ray dataset, further investigation using datasets from other medical modalities is still needed to understand more about the general effect of the regularization. The third limitation was the number of predicted classes. We focused on a binary classification task in this study, but there are medical imaging problems involving multi-class classification tasks. Verifying the effectiveness of our approach in multi-class problems would be an important future work. Another limitation was the assumption of the conditional independence of readers’ biases on the input images, given the true latent variable. For example, for the task of classifying digit 3 and digit 8, some images of digit 3 might look more like digit 8 than other images for some readers, but not for others. For further improvement, the dependence of readers’ biases on the input images could be taken into consideration [12].

### 4.4. Recommendation for Amending Biases

In real-world applications where multiple readers annotate a dataset and the PSDR loss-based method is applied to train ML models, it is recommended to sample a small portion of data points from the whole dataset and obtain readers’ consensus on the samples. This dataset can be used as a test set to see how far it is from the consensus.

This study focused on a regularized loss function in a binary classification task to reduce the effect of biased labels on ML model training. From a methodological viewpoint, there are some other approaches to deal with labeling errors, including transition matrix, robust losses, sample weighting, sample selection, meta-learning, and their combination [11]. In addition to binary classification tasks, there are many other important tasks in medical imaging that ML contributes to (e.g., multi-class classification and segmentation tasks). Assessment of these methodologies and their effectiveness for various ML tasks are important for future work.

## 5. Conclusions

In conclusion, this study demonstrated that it is possible for machine learning algorithms to overcome individual readers’ biases in training a binary classifier. The performance of the classifier was investigated with the introduction of artificial and random biases among multiple readers to the training dataset. We found that a regularized loss function was effective in alleviating the degradation of classification performance caused by the introduced biases. These findings need to be verified using a real dataset where biases are related to reader training and experience.

## Figures and Tables

**Table 1:** Strengths and types of biased and erroneous labels introduced to the dataset. fp: false positive labels. fn: false negative labels. Random error contains a mixture of fp and fn labels because a set of labels containing a mixture of different types of systematic error may be viewed as containing random error.

Error Type		% Level of Introduced Error				
		5%	10%	15%	20%	25%
Random error		5% each	10% each	15% each	20% each	25% each
Systematic error	False positive labeling	10% fp	20% fp	30% fp	40% fp	50% fp
	False negative labeling	10% fn	20% fn	30% fn	40% fn	50% fn

## Data Availability

The dataset used in this work is publicly available. All codes written in support of this publication are publicly available at: https://github.com/7hestral/ImperfectGoldStandard (accessed on 15 April 2023).

## Supplementary Materials

The following supporting information can be downloaded at: https://www.mdpi.com/article/10.3390/tomography9030074/s1. Table S1: Descriptive statistics (mean ± sd) of the models trained using different strengths and types of biases.
