# Supplementary material for: Can Machine Learning Be Better than Biased Readers?
Source: Tomography. 2023 Apr 28;9(3):901–8. doi: 10.3390/tomography9030074 (PMC10204355; doi:10.3390/tomography9030074)
Supplement: Supplementary file 1 [file tomography-09-00074-s001.zip › tomography-2336631-SI.pdf]

Supplementary Table S1. Descriptive statistics (mean ± sd) of the models trained using different strengths and types of biases. Bias_fn: false negative bias introduced to the training set as described in Table 1. Bias_fp: false positive bias introduced to the training set as described in Table 1. Random: random error introduced to the training set as described in Table 1. Baseline: baseline model. Regularized: improved model with regularization applied while training.

% Level of Introduced Error	Error type	Model	Accuracy	AUC	Number of true positives	Number of true negatives	Number of false negatives	Number of false positives
0	/	Baseline	0.8376 ± 0.0142	0.794 ± 0.0199	377.6667 ± 1.8559	145 ± 10.0167	12.3333 ± 1.8559	89 ± 10.0167
		Regularized	0.8584 ± 0.0152	0.8295 ± 0.0235	368.6667 ± 5.3333	167 ± 13.5769	21.3333 ± 5.3333	67 ± 13.5769
5	Bias_fn	Baseline	0.8128 ± 0.0126	0.7974 ± 0.0191	335 ± 6.6408	172.2 ± 11.5213	55 ± 6.6408	61.8 ± 11.5213
		Regularized	0.8446 ± 0.0185	0.8138 ± 0.0287	365.4 ± 5.938	161.6 ± 16.5487	24.6 ± 5.938	72.4 ± 16.5487
	Bias_fp	Baseline	0.8109 ± 0.0109	0.7605 ± 0.0161	375.2 ± 5.1711	130.8 ± 9.4414	14.8 ± 5.1711	103.2 ± 9.4414
		Regularized	0.8625 ± 0.007	0.8464 ± 0.0022	355.2 ± 11.8042	183 ± 7.5697	34.8 ± 11.8042	51 ± 7.5697
	Random	Baseline	0.8128 ± 0.0068	0.7756 ± 0.0082	360.6 ± 4.9051	146.6 ± 4.9457	29.4 ± 4.9051	87.4 ± 4.9457
		Regularized	0.8683 ± 0.0059	0.8447 ± 0.0084	366.2 ± 4.4542	175.6 ± 5.5191	23.8 ± 4.4542	58.4 ± 5.5191
10	Bias_fn	Baseline	0.7721 ± 0.016	0.7739 ± 0.0165	299 ± 6.7231	182.8 ± 5.1711	91 ± 6.7231	51.2 ± 5.1711
		Regularized	0.8423 ± 0.007	0.819 ± 0.0171	355.8 ± 10.5612	169.8 ± 14.0656	34.2 ± 10.5612	64.2 ± 14.0656
	Bias_fp	Baseline	0.8138 ± 0.0161	0.7722 ± 0.0221	366 ± 2.5495	141.8 ± 11.0336	24 ± 2.5495	92.2 ± 11.0336
		Regularized	0.8288 ± 0.0171	0.7874 ± 0.0272	371.8 ± 5.8344	145.4 ± 16.0019	18.2 ± 5.8344	88.6 ± 16.0019
	Random	Baseline	0.7837 ± 0.0087	0.7534 ± 0.0096	341 ± 5.099	148 ± 4.7223	49 ± 5.099	86 ± 4.7223
		Regularized	0.8135 ± 0.0247	0.768 ± 0.0378	370.4 ± 7.3389	137.2 ± 21.3832	19.6 ± 7.3389	96.8 ± 21.3832

15	Bias_fn	Baseline	0.7282 ± 0.0184	0.7467 ± 0.0168	262.4 ± 11.0481	192 ± 5.3759	127.6 ± 11.0481	42 ± 5.3759
		Regularized	0.8494 ± 0.011	0.8397 ± 0.0206	342.6 ± 7.1666	187.4 ± 13.8838	47.4 ± 7.1666	46.6 ± 13.8838
	Bias_fp	Baseline	0.7599 ± 0.0069	0.6946 ± 0.0095	372.8 ± 2.1541	101.4 ± 4.8642	17.2 ± 2.1541	132.6 ± 4.8642
		Regularized	0.8417 ± 0.0077	0.805 ± 0.0152	371.2 ± 6.8659	154 ± 10.9453	18.8 ± 6.8659	80 ± 10.9453
	Random	Baseline	0.7529 ± 0.0056	0.7271 ± 0.0028	323.8 ± 8.0833	146 ± 5	66.2 ± 8.0833	88 ± 5
		Regularized	0.8484 ± 0.0108	0.8235 ± 0.0167	360 ± 6.9065	169.4 ± 10.6986	30 ± 6.9065	64.6 ± 10.6986
20	Bias_fn	Baseline	0.6548 ± 0.0117	0.6934 ± 0.0079	210.2 ± 10.9973	198.4 ± 5.1827	179.8 ± 10.9973	35.6 ± 5.1827
		Regularized	0.8474 ± 0.0039	0.8333 ± 0.01	347 ± 6.5955	181.8 ± 8.4817	43 ± 6.5955	52.2 ± 8.4817
	Bias_fp	Baseline	0.7522 ± 0.012	0.6859 ± 0.0158	371 ± 3.1623	98.4 ± 7.6328	19 ± 3.1623	135.6 ± 7.6328
		Regularized	0.8054 ± 0.0269	0.756 ± 0.041	372 ± 7.1554	130.6 ± 22.936	18 ± 7.1554	103.4 ± 22.936
	Random Random	Baseline	0.7032 ± 0.0051	0.6771 ± 0.0081	304.8 ± 7.9524	134 ± 7.6616	85.2 ± 7.9524	100 ± 7.6616
		Regularized	0.8381 ± 0.0201	0.8122 ± 0.0325	357.2 ± 8.2668	165.8 ± 19.5765	32.8 ± 8.2668	68.2 ± 19.5765
25	Bias_fn	Baseline	0.6144 ± 0.0158	0.6599 ± 0.0109	186.4 ± 13.178	197 ± 4.7749	203.6 ± 13.178	37 ± 4.7749
		Regularized	0.7333 ± 0.0376	0.7725 ± 0.0255	240.2 ± 29.5456	217.4 ± 7.1944	149.8 ± 29.5456	16.6 ± 7.1944
	Bias_fp	Baseline	0.749 ± 0.0098	0.6768 ± 0.0133	376.6 ± 2.7857	90.8 ± 6.6888	13.4 ± 2.7857	143.2 ± 6.6888
		Regularized	0.8054 ± 0.0259	0.7522 ± 0.0379	376.4 ± 6.063	126.2 ± 20.4436	13.6 ± 6.063	107.8 ± 20.4436
	Random	Baseline	0.6724 ± 0.0123	0.6528 ± 0.0157	285.2 ± 4.2237	134.4 ± 7.5206	104.8 ± 4.2237	99.6 ± 7.5206
		Regularized	0.8304 ± 0.0106	0.8158 ± 0.008	341 ± 16.9145	177.2 ± 11.8634	49 ± 16.9145	56.8 ± 11.8634
